# Association between *JAK2* mutations and thoracic aortic aneurysms in myeloproliferative neoplasms

**DOI:** 10.1007/s11239-026-03280-w

**Published:** 2026-05-19

**Authors:** Mohamad Wajeh Dulli, Ashraf Ahmed, Mohammad Altermanini, Rasha Kaddoura, Abdulrahman F. Al-Mashdali, Mohammed Abdulgayoom, Yahia Imam, Shehab F. Mohamed

**Affiliations:** 1 Department of Internal Medicine, Hamad Medical Corporation, Doha, Qatar; 2 Department of Internal Medicine, Yale School of Medicine, Bridgeport Hospital, Bridgeport, CT USA; 3 Department of Cardiology, Heart Hospital, Hamad Medical Corporation, P.O. Box 3050, Doha, Qatar; 4 Department of Pharmacy, Heart Hospital, Hamad Medical Corporation, Doha, Qatar; 5 Department of Hematology, NCCCR, Hamad Medical Corporation, Doha, Qatar

**Keywords:** JAK2 V617F mutation, Myeloproliferative neoplasms (MPNs), Aortic aneurysm, Cardiovascular risk

## Abstract

The Janus kinase 2 (JAK2) V617F mutation is recognized as a defining molecular lesion in Philadelphia chromosome-negative myeloproliferative neoplasms (MPNs). This mutation was recognized in the context of clonal hematopoiesis of indeterminate potential, which has recently been linked with cardiovascular disease. Recent evidence has demonstrated a significant correlation between JAK2 mutations and thoracic aortic aneurysm (TAA) a condition frequently lacking identifiable risk factors**.** A scoping review, involving MEDLINE, Embase, Web of Science, Google Scholar, and prominent hematology and cardiology conference abstracts (from inception to August 2025) was performed. All studies that indicate a correlation between JAK2 mutations and aortic aneurysms were included. Seven studies with a total of 446,839 participants were found. Among these, 745 (0.17%) tested positive for JAK2. Data consistently indicated an elevated risk of thoracic aortic aneurysm (TAA), but not abdominal aortic aneurysm, in individuals with JAK2 mutations. Proposed mechanisms include inflammatory cytokine signaling, macrophage activation, and degradation of the extracellular matrix. Emerging clinical data indicate a consistent association between JAK2 V617F–mutated clonal hematopoiesis or myeloproliferative neoplasms and thoracic aortic aneurysm, with less consistent findings for abdominal aneurysm. Although experimental studies suggest that JAK2-driven inflammatory signaling and extracellular matrix remodeling may contribute to vascular wall injury, these mechanisms remain largely inferential and do not establish causality. Well-designed prospective investigations incorporating standardized imaging, uniform aneurysm definitions, and stratification by clonal burden and disease context will be necessary to better define risk and determine whether JAK2-associated clonal hematopoiesis has implications for vascular surveillance or prevention strategies.

**Graphical abstract:**

Summary of the study design, key results, and conclusions. Created with assistance from OpenAI ChatGPT (DALL·E image generation tool); all scientific content was reviewed and verified by the authors.

**Figure:**
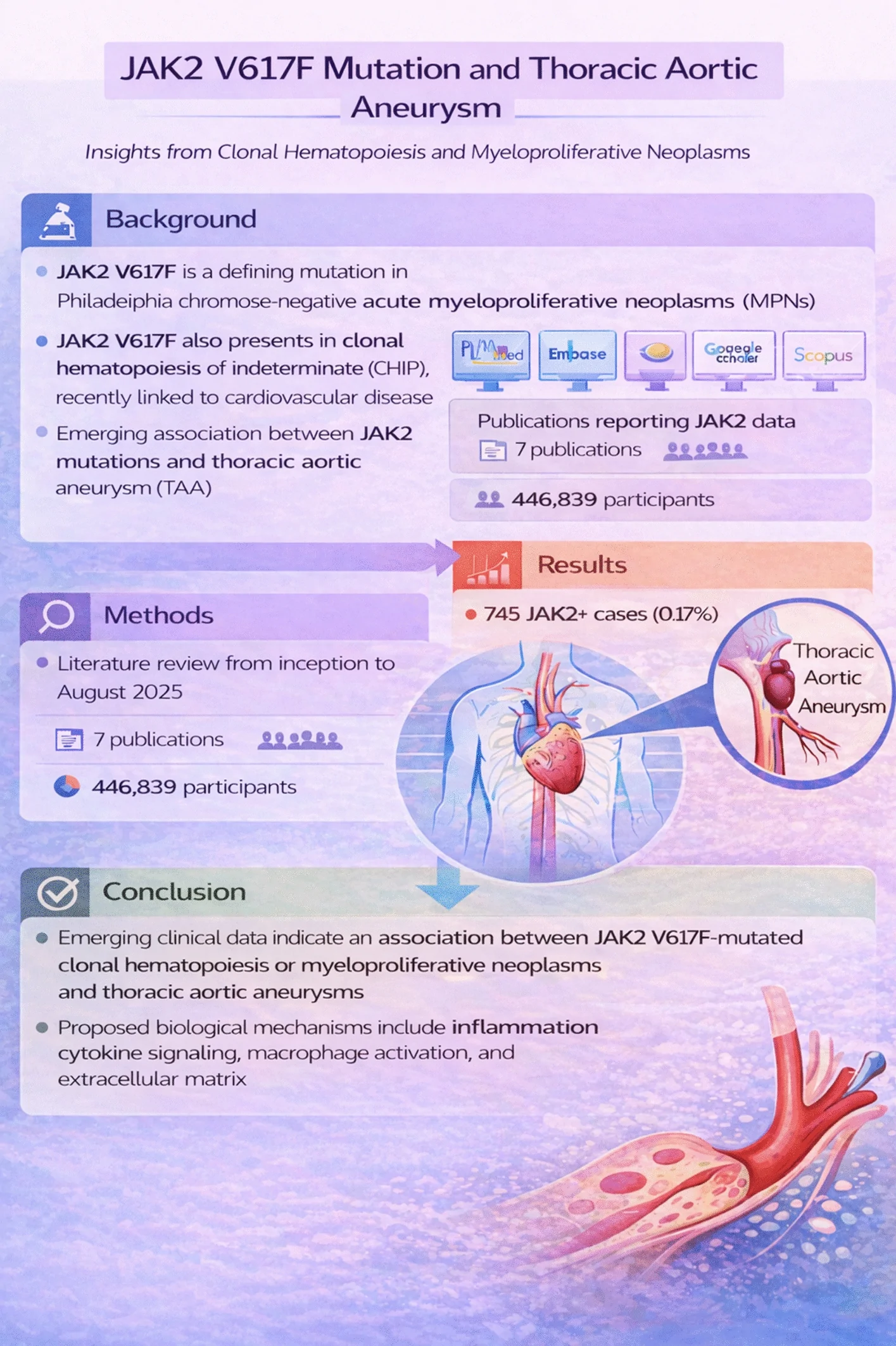

## Introduction

The Janus kinase 2 (*JAK2)* V617F mutation is a somatic, gain-of-function variant that results in constitutive activation of the *JAK*-STAT signaling pathway. It is widely recognized as a defining molecular lesion in Philadelphia chromosome-negative myeloproliferative neoplasms (MPNs), including polycythemia vera (PV), essential thrombocythemia (ET), and primary myelofibrosis (PM) [1, 2]. This mutation leads to abnormal proliferation and survival of hematopoietic progenitor cells, primarily affecting the myeloid lineage. Approximately 95% of patients with PV and 50–60% of those with ET or PM harbor this mutation [3].

More recently, the *JAK2* V617F mutation has gained attention in the context of clonal hematopoiesis of indeterminate potential (CHIP), a condition marked by the presence of hematologic driver mutations in the absence of overt malignancy, particularly in older adults [4]. CHIP is associated with increased all-cause mortality and is now firmly linked to cardiovascular disease, including myocardial infarction, stroke, and heart failure [5, 6]. Among CHIP-related mutations (e.g., *DNMT3A and TET2*), *JAK2* V617F appears to carry the strongest cardiovascular risk, likely due to its pro-inflammatory effects on the vascular endothelium and resident immune cells [7].

Recent research has revealed a striking and previously underappreciated link between *JAK2* V617F and thoracic aortic aneurysm (TAA). TAAs are progressive dilations of the thoracic segment of the aorta and carry a high risk of rupture or dissection, both of which are frequently fatal [8]. While syndromic conditions such as Marfan syndrome or bicuspid aortic valve disease account for a fraction of cases, a significant proportion of TAAs remains idiopathic [8]. Emerging evidence now suggests that somatic mutations in hematopoietic cells, particularly *JAK2* V617F, may represent a novel, non-traditional risk factor for aortic aneurysm development [9]. This association appears to be location-specific, as multiple human cohort studies have shown a significantly increased risk of thoracic, but not abdominal, aortic aneurysms in individuals with *JAK2* V617F-positive CHIP or MPNs [10]. The mutation’s effect seems to be mediated through pro-inflammatory cytokine signaling, monocyte/macrophage activation, and extracellular matrix degradation, leading to weakening of the aortic wall [11].

Given the clinical implications of this mutation in aortic pathology, it is crucial to understand the mechanisms underlying *JAK2* V617F-induced vascular injury and to assess its potential role in screening and early intervention strategies. This review synthesizes the mechanistic and clinical data connecting *JAK2* V617F to TAA risk, highlighting both experimental insights and population-based evidence**.**

## Methods

A search of the medical literature was conducted by two independent researchers to identify publications reporting the association between *JAK2* mutations and aortic aneurysms. We performed an online search of three databases, MEDLINE (PubMed), Embase, and Web of Science, using Boolean operators, key words, and MeSH/EMTREE terms. The search strategy included combinations of terms such as “*JAK2* V617F”, “exon 12 JAK2”, “*CALR* mutation”, “*MPL* mutation”, “myeloproliferative neoplasms”, “clonal hematopoiesis”, “thoracic aortic aneurysm”, and “abdominal aortic aneurysm”. We also searched Google Scholar for additional articles. The search duration of these databases was from inception to August 1, 2025. All articles which reported the *JAK2* mutation and aortic aneurysm association was included regardless of the study design. Only English-language papers were included. Titles and abstracts of the literature search results were screened. Full-text of potential articles that reported patient-level data or aggregated outcomes related to *JAK2* mutations and aortic aneurysms were retrieved. In addition, abstracts from the American Society of Hematology (ASH), European Hematology Association (EHA), and American College of Cardiology (ACC) annual meetings were reviewed to capture unpublished or recent data. Finally, reference lists of retrieved articles were hand-searched to identify additional relevant reports missed during the initial search.

## Results

We identified seven articles encompassing a total of 446,839 participants screened for aortic aneurysms, of whom at least 745 (0.17%) were *JAK2-*positive. The studies provided subgroup data on thoracic and abdominal aneurysms related to *JAK2* mutation (Table 1).

**Table 1:** Summary of human studies on JAK2 V617F and aneurysm risk

Study	Type of the study	Number of subjects screened	*JAK2* + mutated	Thoracic aneurysm cases	Abdominal aneurysm cases	Key findings
[8]	Retrospective cohort study	417,759	118	4	1	*JAK2* V617F CHIP associated with 12.8 × risk for TAA; no association with AAA
[14]	Descriptive study	12,439	12	5	0	*JAK2* V617F somatic variant may play a significant role in the development of thoracic aortic aneurysm
[13]	Descriptive study	39	39	7	2	*JAK2* V617F enhance aneurysms formation via inflammatory mechanisms
[10]	Retrospective cohort study	382	382	157 patients had significant dilated ascending, descending thoracic or abdominal aorta		*JAK2* V617F associated with larger thoracic and descending aortic diameters
[9]		8,056	570	54	NA	*JAK2* V617F VAF ≥ 1% strongly associated with increased TAA risk
[15]	Descriptive study	492	6	3	NA	There is evidence for a possible link between *JAK2-*V617F and TAA

AAA: Abdominal aortic aneurysms; JAK-2: Janus kinase 2; TAA: thoracic aortic aneurysm; VAF: variant allele frequency

### Thoracic aortic aneurysms (TAA)

Across all included studies, a consistent association was observed between *JAK2* positivity and thoracic aortic aneurysms. In total, 73 thoracic aneurysms were reported among *JAK2*‑positive patients, compared with 1,115 cases among *JAK2*‑negative individuals. In the largest retrospective cohort including 417,759 participants, Nakao et al. reported that 118 patients with *JAK2*‑positive, of whom 4 (2.9%) had thoracic aneurysms [8]. The risk of thoracic aneurysm in this population was markedly elevated, with a hazard ratio of 12.8 (95% CI: 4.8–34.0; P = 3.6 × 10⁻⁷) [8]. Similarly, in a cross‑sectional cohort of 8,056 individuals, 570 (7.1%) were identified as *JAK2-*positive of whom thoracic aneurysms were observed in 9% of those with a low variant allele frequency (VAF; < 1%) and in 16.5% of those with a high VAF (≥ 1%) [9]. Hazard ratios confirmed a dose‑response relationship, measuring 1.4 (95% CI: 1.01–2.0) for low VAF and 2.7 (95% CI: 1.5–5.1) for high VAF [9]. Moreover, other descriptive studies in a smaller scale demonstrated the same association highlighted in Table 1.

### Abdominal aortic aneurysms (AAA)

On the other hand, evidence linking *JAK2* positivity to abdominal aortic aneurysms ware limited and inconsistent. Across all the included studies, at least 3 abdominal aneurysm cases were reported in *JAK2*‑positive patients. In the largest population cohort included in this review, only a single case of abdominal aneurysm was observed among *JAK2‑*positive participants [8]. Moreover, Yokokawa et.al reported two patients with abdominal aortic aneurysm in their cohorts out of study of 39 patients with *JAK2* mutation. Waldron et.al reported a zero AAA in their cohorts of 12,439 patients (Table 1).

## Discussion

### JAK2 V617F and vascular risk beyond hematologic malignancy

Beyond hematologic malignancies, *JAK2 V617F* has been associated with worse cardiovascular outcomes, including increased risk of arterial and venous thrombosis (deep vein thrombosis, pulmonary embolism, myocardial infarction, ischemic stroke), even in individuals without overt MPN. This broader vascular phenotype is also relevant in the setting of clonal hematopoiesis of indeterminate potential (CHIP), where *JAK2*-driven clones may confer a vascular risk profile approaching that observed in overt *JAK2*-mutated MPNs despite the absence of cytoses or a formal hematologic diagnosis. In a large biobank analysis (~ 54,000 individuals), *JAK2*-CHIP carriers (median VAF ~ 16%) demonstrated thrombotic event rates that were broadly comparable to patients with *JAK2*-mutated MPNs, and cardiovascular complications extended beyond thrombosis to include arrhythmias and heart failure. Importantly, many venous thrombotic events in MPN cohorts occurred prior to or within two years of diagnosis, suggesting vascular complications may arise early during clonal expansion. Collectively, these observations support the concept that *JAK2*-CHIP represents a clinically meaningful cardiovascular risk factor, and provide a biologically plausible context for examining TAA risk in *JAK2* carriers. However, the degree to which these associations translate into actionable aneurysm-specific management remains uncertain in the absence of prospective evidence.

### Mechanisms and pathophysiology (hypotheses informed largely by preclinical evidence)

Mechanistic work from murine models and selected human tissue analyses provides a plausible framework linking *JAK2 V617F* to thoracic aortic pathology, but these pathways should be interpreted primarily as hypotheses rather than clinically proven mechanisms [12]. Current models propose that the mutation originates in the hematopoietic stem cell compartment, favoring myeloid-biased clonal expansion and generating circulating monocytes/macrophages with altered inflammatory signaling. These cells may infiltrate the aortic wall and adopt a pro-inflammatory phenotype, producing cytokines such as IL-1β, IL-6, and TNF-α and activating downstream pathways (including STAT-family signaling) that can promote vascular remodeling. In these models, increased expression of matrix metalloproteinases (notably MMP-2 and MMP-9) contributes to extracellular matrix degradation (elastin/collagen) and progressive weakening of the vessel wall.

Anatomical “thoracic specificity” has been proposed, potentially related to regional differences in hemodynamic stress, wall structure, and immune cell distribution; however, this remains speculative and has not been definitively validated in humans. Preclinical interventional data suggest that pharmacologic inhibition of JAK signaling (e.g., ruxolitinib in animal models) can reduce inflammatory signaling and mitigate aneurysm development, supporting biological plausibility [15]. Nonetheless, it is premature to extrapolate these findings to clinical prevention strategies in humans without dedicated trials.

### Human studies (observational association with thoracic—but not abdominal—aneurysm)

Across multiple human datasets—population-level biobank analyses, cardiovascular screening cohorts, and imaging-enriched MPN cohorts*—JAK2* V617F is repeatedly associated with higher TAA prevalence/risk, while an analogous relationship with abdominal aortic aneurysm (AAA) is not consistently observed. In UK Biobank-derived analyses, JAK2 V617F carriers were rare but demonstrated a markedly elevated relative risk for thoracic aneurysm compared with non-carriers, including carriers without known hematologic malignancy. In the DANCAVAS cardiovascular screening trial, *JAK2* V617F carriers (especially those with higher VAF thresholds) showed increased prevalence of ascending TAA compared with non-carriers, whereas AAA rates were similar. Smaller exome-sequencing cohorts and cross-sectional MPN imaging studies similarly reported a disproportionate burden of thoracic aneurysm or increased thoracic aortic diameters among *JAK2-*positive individuals [13–16].

While these convergent findings strengthen the consistency of the association, key limitations persist: imaging ascertainment varies across studies; the definition of aneurysm and reporting of aortic diameters are not uniform; and confounding by age, comorbidities, and health system contact remains difficult to fully control. Therefore, current human evidence supports association, not causation, and does not define the optimal approach to clinical management.

### Beyond aortic aneurysm: aortic dissection and other arterial dissections (emerging signal)

A potential extension of this phenotype is a predisposition to aortic dissection and other forms of arterial dissection. Preclinical work suggests that *JAK2* V617F-mutant hematopoietic cells can drive inflammatory macrophage phenotypes, elevate cytokine production, and increase metalloproteinase activity, contributing to progressive wall weakening and dissecting aneurysm phenotypes in murine JAK2-CHIP carriers demonstrated thrombotic event ratesmodels [17]. Importantly, these findings provide mechanistic plausibility but remain preclinical and should not be interpreted as evidence of a proven clinical pathway.

Clinically, the spectrum of vascular complications in *JAK2*-mutated disease may include arterial dissections beyond the aorta. Emerging case-based literature has reported spontaneous coronary artery dissection (SCAD) in patients with JAK2 V617F-positive MPNs [18]. Such reports expand the hypothesized vascular fragility phenotype but remain limited by small numbers and publication bias. Large, well-phenotyped cohorts are needed to determine incidence, attributable risk, and the extent to which these complications are independent of traditional atherosclerotic risk factors.

### Beyond JAK2 V617F: other driver mutations

Although JAK2 V617F has the strongest and most reproducible association with thrombotic and vascular outcomes, analogous evidence for *JAK2* exon 12, *CALR*, or MPL mutations is limited. Some data suggest lower thrombotic risk in *CALR-*mutated ET compared with *JAK2*-mutated ET, and no clear thrombotic differences for *JAK2* exon 12 compared to V617F in PV in certain datasets [19, 20]. These observations raise the hypothesis that non–*JAK2* V617F drivers may confer a less pronounced inflammatory/thrombotic phenotype, potentially explaining the lack of reported aneurysm associations to date; however, absence of evidence is not evidence of absence, and systematic evaluation in large cohorts is still needed.

### Implications and research priorities

This review consolidates observational signals linking *JAK2* V617F (in both CHIP and overt MPN contexts) with thoracic aortic pathology and integrates preclinical mechanisms that may explain biological plausibility. However, given the observational nature of most human studies and the potential for confounding and detection bias, the primary implications should be framed as research priorities rather than immediate changes to clinical practice.

### Strength of evidence, confounding, and potential detection bias

The available evidence supporting an association between *JAK2* V617F and thoracic aortic aneurysm (TAA) is increasingly consistent across cohorts; however, it is important to emphasize that most human data are observational (cross-sectional analyses, retrospective cohorts, and biobank-based studies). As such, these studies can demonstrate association, but do not establish causality. Several sources of bias may influence effect size estimates, including residual confounding (age, smoking, hypertension, dyslipidemia, body size, connective tissue risk factors, and medication exposure), and detection bias. Individuals with known or suspected clonal hematopoiesis or MPNs may undergo more frequent clinical evaluation and imaging for cardiovascular symptoms or thrombotic events, which could increase the likelihood of identifying incidental aortic dilation or aneurysm. Additionally, cohorts differ in imaging modalities, thresholds used to define aneurysm, and whether outcomes reflect “diagnosed aneurysm” versus measured diameter changes, limiting direct comparability. These limitations require a cautious interpretation of clinical implications and support the need for prospective validation.

### Key perspectives

Given that most human evidence linking *JAK2* V617F to thoracic aortic aneurysm is observational and potentially influenced by residual confounding and detection bias, the most appropriate next step is to define the association prospectively using standardized imaging protocols and pre-specified aneurysm outcomes. Future studies should explicitly distinguish overt MPN from JAK2-driven CHIP, incorporate variant allele fraction (VAF**)** and clonal dynamics as continuous risk markers, and clarify whether risk scales with clone size or duration of clonal expansion. Equally important is determining the real-world imaging yield in *JAK2* carriers, including the prevalence of clinically meaningful thoracic dilation at baseline, rates of progression over time, and the frequency of hard endpoints such as surgery, dissection, rupture, or aneurysm-related mortality. These prospective data should then inform formal cost-effectiveness and decision-analytic models to evaluate whether any targeted imaging approach could be justified, and in which subgroups, rather than assuming benefit. Finally, translational work that links circulating inflammatory signatures and myeloid phenotypes to human aortic remodeling—ideally with paired imaging and biomarker sampling—will be critical to bridge the gap between preclinical mechanistic hypotheses (e.g., macrophage-driven inflammation and MMP-mediated matrix degradation) and clinically validated pathways in humans.

In summary, *JAK2* V617F appears to be associated with a thoracic aortic aneurysm signal across multiple cohorts, supported by mechanistic plausibility from preclinical studies. Nonetheless, causality and optimal clinical actions remain unproven, and future work should focus on prospective risk definition and the clinical utility of imaging approaches.

### Limitations

Several limitations should be acknowledged. The available evidence is predominantly observational, and therefore supports association rather than causation. The included studies were heterogeneous in design, patient population, aneurysm definition, imaging strategy, and outcome reporting**,** limiting direct comparison across cohorts. In addition, residual confounding and detection bias are important concerns, as patients with known MPN or JAK2-mutated clonal hematopoiesis may undergo more frequent imaging and clinical surveillance than unaffected individuals. Longitudinal imaging and hard clinical outcome data were limited, making it difficult to assess aneurysm progression, dissection, rupture, or need for intervention. Finally, mechanistic support is based largely on preclinical data**,** which provides biologic plausibility but does not establish clinically validated causal pathways in humans. These limitations underscore the need for prospective studies.

## Conclusion

The current literature suggests an observational association between JAK2 V617F and thoracic aortic aneurysm**,** supported by both clinical datasets and mechanistic studies. However, available evidence remains heterogeneous and largely observational, and causal inference cannot be established. At present, these findings do not justify routine vascular screening based on JAK2 mutation status**.** Prospective studies with standardized imaging and detailed clonal characterization will be necessary to define the clinical relevance of this association.

## Data Availability

The datasets used and/or analyzed during the current study are available from the corresponding author on request.
